# Neuroprotective Effect of Salvianolic Acid A against Diabetic Peripheral Neuropathy through Modulation of Nrf2

**DOI:** 10.1155/2020/6431459

**Published:** 2020-02-27

**Authors:** Chunyang Xu, Biyu Hou, Ping He, Peng Ma, Xinyu Yang, Xiuying Yang, Li Zhang, Guifen Qiang, Wenlan Li, Guanhua Du

**Affiliations:** ^1^State Key Laboratory of Bioactive Substance and Function of Natural Medicines, Institute of Materia Medica, Chinese Academy of Medical Sciences and Peking Union Medical College and Beijing Key Laboratory of Drug Target and Screening Research, Beijing 100050, China; ^2^College of Pharmacy, Harbin University of Commerce, Harbin 150076, China; ^3^Beijing Obstetrics and Gynecology Hospital, Capital Medical University, Beijing 100006, China; ^4^College of Pharmacy, Guangdong Medical University, Dongguan 523808, China

## Abstract

Oxidative stress has been recognized as the contributor to diabetic peripheral neuropathy (DPN). Antioxidant strategies have been most widely explored; nevertheless, whether antioxidants alone prevent DPN still remains inconclusive. In the present study, we established an *in vitro* DPN cell model for drug screening using Schwann RSC96 cells under high glucose (HG) stimulation, and we found that salvianolic acid A (SalA) mitigated HG-induced injury evidenced by cell viability and myelination. Mechanistically, SalA exhibited strong antioxidative effects by inhibiting 1,1-diphenyl-2-picrylhydrazyl (DPPH) and reducing reactive oxygen species (ROS), malondialdehyde (MDA), and oxidized glutathione (GSSG) content, as well as upregulating antioxidative enzyme mRNA expression. In addition, SalA significantly extenuated neuroinflammation with downregulated inflammatory factor mRNA expression. Furthermore, SalA improved the mitochondrial function of HG-injured Schwann cells by scavenging mitochondrial ROS, decreasing mitochondrial membrane potential (MMP), and enhancing ATP production, as well as upregulating oxidative phosphorylation gene expression. More importantly, we identified nuclear factor-E2-related factor 2 (Nrf2) as the upstream regulator which mediated protective effects of SalA on DPN. SalA directly bound to the Kelch domain of Kelch-like ECH-associated protein 1 (Keap1) and thus disrupted the interaction of Nrf2 and Keap1 predicted by LibDock of Discovery Studio. Additionally, SalA significantly inhibited Nrf2 promoter activity and downregulated Nrf2 mRNA expression but without affecting Nrf2 protein expression. Interestingly, SalA upregulated the nuclear Nrf2 expression and promoted Nrf2 nuclear translocation by high content screening assay, which was confirmed to be involved in its antiglucotoxicity effect by the knockdown of Nrf2 in RSC96 cells. In KK-Ay mice, we demonstrated that SalA could effectively improve the abnormal glucose and lipid metabolism and significantly protect against DPN by increasing the mechanical withdrawal threshold and sciatic nerve conduction velocity and restoring the ultrastructural impairment of the injured sciatic nerve induced by diabetes. Hence, SalA protected against DPN by antioxidative stress, attenuating neuroinflammation, and improving mitochondrial function via Nrf2. SalA may be prospective therapeutics for treating DPN.

## 1. Introduction

Diabetic peripheral neuropathy (DPN), the most common microvascular complications of diabetes mellitus, affects approximately 50% of patients over the course of diseases with high morbidity and mortality [1]. Apparently, metabolic imbalances are closely associated with the risk of developing DPN, such as hyperglycemia, dyslipidemia, and cardiovascular dysfunction [2]. Moreover, accumulation of sorbitol, formation of advanced glycation end products (AGEs), oxidative stress, and neuroinflammation are also involved in the molecular mechanism [3, 4].

Oxidative stress has been recently recognized as the final common pathway of cellular injury under hyperglycemic condition [5]. Long-term persistence of hyperglycemia leads to excess neuronal glucose uptake and inadequate disposal of intracellular glucose by glycolysis. Raised intracellular glucose subsequently drives the alternative polyol pathway contributing to oxidative stress and mitogen-activated protein kinase (MAPK) activation, which is defined as glucose neurotoxicity [6]. The hyperglycemia-induced injury in neurons, Schwann cells, and microvascular endothelial cells contributes to the pathogenesis of neuropathy with loss of protective sensation, particularly in the feet at the end stage. Nuclear factor-E2-related factor 2 (Nrf2) belongs to the family of transcription factors regulating a set of antioxidants and detoxification enzymes. It was reported that Nrf2 acted as a bridge linking oxidative stress and inflammation and impacted progression of diabetic neuropathy, which has been demonstrated by treatment with Morin and alpha lipoic acid [7, 8]. Antioxidant strategies have been most widely explored; nevertheless, whether antioxidants alone prevent DPN still remains inconclusive since the molecular mechanisms are more complex.

Salvianolic acid A (SalA) ((2R)-3-(3,4-dihydroxyphenyl)-2-[a-(3,4-dihydroxyphenyl) prop-2-enoyloxy] propanoic acid) is one of the main active water-soluble components of *Salvia miltiorrhiza* [9]. It has been demonstrated that SalA possessed extensive pharmacological activities including antioxidation, antiplatelet aggregation, and anti-ischemia [10–12]. Furthermore, SalA exerted antidiabetic effects and protected against diabetic complications such as diabetic nephropathy, diabetic periphery neuropathy, and diabetes with hepatic fibrosis as well [13–16]. Our previous studies have shown that salvianolic acid A protected peripheral nerve function in type 1 diabetic rats through the AMPK-PGC-1a-Sirt3 signaling pathway [16]. As of yet, however, it is still unknown whether SalA likely improves diabetic periphery neuropathy through attenuating oxidative stress since SalA is a strong antioxidant.

In the current study, we established an *in vitro* DPN cell model for drug screening and evaluation using rat Schwann cells under high glucose challenge and evaluated the protective effects of SalA on high glucose-induced injury in RSC96 cells. Then, we further verified the protection of SalA on diabetic peripheral neuropathy in KK-Ay diabetic mice by assessing behavior and ultrastructural changes of the sciatic nerve. Finally, we investigated the underlying mechanisms of attenuating oxidative stress and neuroinflammation induced by high glucose injury and subsequently improving mitochondrial function in Schwann cells through modulation of Nrf2.

## 2. Materials and Methods

### 2.1. Reagent and Chemicals

Salvianolic acid A was provided by the Institute of Materia Medica, Chinese Academy of Medical Sciences, as a lyophilized powder withpurity > 98%. Sulforhodamine B (SRB) and 1,1-diphenyl-2-picrylhydrazyl (DPPH) were obtained from Sigma, USA. Hoechst 33342 was obtained from Cell Signaling, USA. Dichlorodihydro-fluorescein diacetate (DCFH-DA) was obtained from APPLYGEN, China. Rhodamine 123 was obtained from MCE, USA.

### 2.2. Antibodies

Anti-nerve growth factor (NGF, YT3114) was obtained from ImmunoWay (USA). Anti-myelin protein zero (MPZ/P0, 10572-1-AP), anti-Nrf2 (16396-1-AP), and anti-Kelch-like ECH-associated protein 1 (Keap1, 60027-1-lg) were obtained from Proteintech (USA). Anti-histone H3 (4499) was obtained from Cell Signaling (USA). Anti-*β*-actin (30101ES60) was obtained from YEASEN (China).

### 2.3. Cell Lines and Cell Culture

The rat Schwann cell line (RSC96) was obtained from the Institute of Basic Medical Sciences, Chinese Academy of Medical Sciences (Beijing, China). The cells were cultured in DMEM media (4.5 g/L glucose) supplemented with 10% fetal bovine serum (FBS), 1% penicillin/streptomycin at 37°C, and 5% CO_2_.

### 2.4. Determination of Cell Viability

RSC96 cells were seeded at 1 × 10^4^ cells/well in a 96-well plate and were treated with various concentrations of glucose for 24 h. Then, the cells were incubated with 25 mM glucose (normal glucose, NG) or 150 mM glucose (high glucose, HG) with or without different concentrations of SalA (0.3, 1, and 3 *μ*M) for 24 h.

*Cell Counting Kit-8 (CCK-8) detection*: the highly water-soluble tetrazolium salt, WST-8 (2-(2-methoxy-4-nitrophenyl)-3-(4-nitrophenyl)-5-(2,4-disulfophenyl)-2H-tetrazolium, monosodium salt), produces orange-colored formazan dye upon reduction by dehydrogenases in cells. The amount of formazan dye is directly proportional to the number of living cells. Briefly, 10 *μ*L CCK-8 (MCE, USA) solution was added and incubated at 37°C for 1 h. Absorbance was measured at 450 nm using a SpectraMax M5 microplate reader (Molecular Devices, USA).

*SRB detection*: SRB dye stoichiometrically binds to proteins under mild acidic conditions and subsequently extracts under basic conditions. The amount of dye extracted is a proxy for cell mass and thus cell number. Briefly, 50 *μ*L of precooled 50% trichloroacetic acid (TCA) was added to fix the cells. After washing 5 times using deionized water, 80 *μ*L of 0.4% SRB in 1% acetic acid per well was added and incubated for 10 min at room temperature. Wash 5 times with 1% acetic acid and add 200 *μ*L of pH 10.5 10 mM Tris. Absorbance was measured at 540 nm by a SpectraMax M5 microplate reader (Molecular Devices, USA).

### 2.5. RNA Extraction and Real-Time PCR

Total RNA was isolated using a TRIzol reagent (Invitrogen, USA) and Direct-zol kit (Zymo, USA). cDNA was prepared from 2 *μ*g of total RNA using the High-Capacity cDNA Reverse Transcription Kit (Applied Biosystems, USA) according to the manufacturer's instructions. The obtained cDNA was diluted, and a 5 *μ*L aliquot was used in a 11 *μ*L PCR reaction (SYBR Green, Vazyme, China) containing a primer at a concentration of 300 nM. The PCR reaction was performed in triplicate and quantified using a Bio-Rad CFX96 qPCR system (Bio-Rad, USA). The results were normalized to a TATA box binding protein (TBP) expression and expressed as arbitrary units or fold changes (see Table 1 for detailed primer sequences).

### 2.6. Animals

8-week male KK-Ay mice and C57BL/6J of SPF grade were purchased from Beijing Huafukang Biotechnology Co. Ltd. The mice were raised in the Experimental Animal Center of the Institute of Materia Medica, Chinese Academy of Medical Sciences at room temperature of 22-25°C, humidity of 40-60%, and a 12 h light/dark cycle to adapt for two weeks. All experimental animals were kept and operated in accordance with the guidelines of the Experimental Animal Ethics Committee of the Institute of Materia Medica, Chinese Academy of Medical Sciences.

### 2.7. Grouping and Administration

After 2-week adaptive feeding, fasting blood glucose (FBG) above 10 mmol/L was considered as the diabetic model. Based on the previous findings of SalA on type 1 and 2 diabetic complications in our lab, we chose SalA 1 mg/kg/d as the treatment dose in our current study [15, 16]. Thus, diabetic mice were randomly divided into two groups, including the diabetic model group (DM group) and the salvianolic acid A 1 mg/kg group (SalA 1 mg/kg group). Another 10 male C57BL/6J mice were the normal control group (NC group). The NC group and DM group were given an equal volume of distilled water, and the SalA 1 mg/kg group was administered at a dose of 1 mg/kg once a day for 8 weeks. Body weight and 24 h food and water intake were recorded. At the end of the 8-week administration, blood samples were collected and the sciatic nerve tissues were immediately frozen in liquid nitrogen or fixed in 10% neutral buffered formalin for histological diagnosis. The sciatic nerve was excised and fixed in 2% paraformaldehyde-2.5% glutaraldehyde for transmission electron microscopy examination.

### 2.8. Measurement of Blood Glucose, Fructosamine, and Glucose Tolerance Test

The fructosamine (total glycated serum proteins) level was determined according to the manufacturer's instruction (BSBE, China). After the mice were fasted for 4 h, FBG was detected using Accu-Chek blood glucose meters (Roche, USA). Glucose tolerance test (GTT) was performed in the animals after fasting for 4 h followed by intraperitoneal injection with glucose solution (2.0 g/kg). The blood glucose level was determined at 0, 30, 60, and 120 min after glucose loading using Accu-Chek blood glucose meters (Roche, USA).

### 2.9. Measurement of Blood Lipids

At the end of the 8-week treatment, blood lipids of triglyceride (TG) and total cholesterol (TC) were detected according to the manufacturer's instruction of the TG and TC test kit (Zhongsheng Beikong Biotechnology Co., Ltd., China) with an automatic analyzer (Toshiba Accute TBA-40FR, Toshiba Corporation, Tokyo, Japan).

### 2.10. Hot Plate Test

A hot plate experiment was performed on the KK-Ay mice during the end-stage treatment to determine the thermal withdrawal latency (TWL) by YLS-6B intelligent hot plate tester (Jinan Yiyan Technology Development Co., Ltd., China). The temperature was set in the hot plate at 52°C. The thermal withdrawal latency is defined as the time since the foot touches on hot plate until paw licking (unit: second).

### 2.11. Measurement of Paw Mechanical Withdrawal Threshold

At the end of the 8-week treatment, mechanical allodynia was measured by assessing mouse hind paw mechanical withdrawal threshold (MWT) in response to mechanical stimulation using an electrical mechanical analgesia tester (BME-404, Institute of Biomedical Engineering Chinese Academy of Medical Sciences, Tianjin, China). The mice were placed in a hanging cage with a metal mesh floor. A mechanical stimulus was applied to the plantar surface of the hind paw by a stainless-steel filament (0.6 mm in diameter) exerting a linearly increasing force. The force (grams) at which paw withdrawal occurred was automatically recorded.

### 2.12. Examination of Blood Flow

The blood flow of foot and ear was examined by the Laser Doppler Flowmeter (PeriFlux500, PERIMED, Sweden). The probes were placed on the flat part of the mouse's foot and ear to gently touch the tissue. Record the value for 1-3 minutes and select the flat and stable curve to read the blood perfusion.

### 2.13. Evaluation of Motor Nerve Conduction Velocity

Motor nerve conductive velocity (MNCV) was examined through a BL-420 biomechanical system (Taimeng, Chengdu, China). Briefly, the left tibial nerve was stimulated at the proximal end with square wave pulses (duration: 0.01 ms, intensity: 1 V) and delivered through bipolar recording electrodes. The action potential was recorded at the distal end. Average of 8 potential traces was recorded by recording the electrode. MNCV (m/s) = (distance between stimulating and recording electrode)/latency.

### 2.14. Immunohistochemistry Examination

The sections were dewaxed in xylene and dehydrated in alcohol. Antigen retrieval was achieved in citric saline by microwaving at 95°C for 3 min, then sections were treated with 3% hydrogen peroxide for 25 min. After blocking with 5% BSA, sections were incubated with primary antibody against rabbit anti-NGF (1 : 500) and anti-P0 antibody (1 : 500) at 4°C overnight followed by incubation with HRP-conjugated goat anti-rabbit IgG (Dako, Wuhan, China) at room temperature for 50 min. NGF and P0 expression was visualized by 3,3′-diaminobenzidine (DAB, Dako, Wuhan, China) staining. Sections were then examined under an optical microscope (Nikon Eclipse Ti-U, Nikon Corporation, Tokyo, Japan) and the photographs were taken.

### 2.15. Transmission Electron Microscopy of Sciatic Nerve

The sciatic nerve was excised and fixed in 2% paraformaldehyde-2.5% glutaraldehyde at 4°C for 2 h. After fixation, the samples were rinsed 3 times with PBS buffer and finally stored in PBS buffer at 4°C. The samples were then dehydrated and embedded in epon. The ultrathin sections cut from the embedded blocks were stained with uranyl acetate and lead citrate and were examined under a transmission electron microscope (TEM17650, Hitachi, Tokyo, Japan). Image-Pro Plus software was used for quantitative ultrastructural analysis. Briefly, nonoverlapping photographs were taken from the transverse sections and an average of 10 microscopic fields per section were analyzed to cover the entire cross-section of the sciatic nerve.

### 2.16. In Vitro DPPH Assay

Fresh DPPH working solution and various concentrations of SalA were incubated at room temperature for 30 min. The absorbance at 515 nm was detected by SpectraMax M5 microplate reader (Molecular Devices, USA).

### 2.17. ROS Detection

The intracellular reactive oxygen species (ROS) content was determined with a peroxide-sensitive fluorescent probe DCFH-DA. Briefly, RSC96 cells were seeded at 1 × 10^4^ cells/well in a 96-well black plate and treated with 25 mM or 150 mM glucose and different concentrations of SalA for 24 h. Then, the cells were harvested and incubated with 10 *μ*M DCFH-DA for 30 min at 37°C. The fluorescence intensity was then analyzed at excitation 488 nm and emission 525 nm using a SpectraMax M5 microplate reader (Molecular Devices, USA).

### 2.18. Determination of ABTS, GSSG, MDA, and SOD

RSC96 cells were seeded at 3 × 10^5^ cells/well in a 6-well plate and incubated with 25 mM or 150 mM glucose and different concentrations of SalA for 24 h. The cells were fully lysed, and the supernatant was taken for detection according to the manufacturer's instruction for 2,2′-azino-bis(3-ethylbenzothiazoline-6-sulphonic acid) (ABTS), oxidized glutathione (GSSG), malondialdehyde (MDA), and superoxidase dismutase (SOD) test kits (Beyotime, China).

### 2.19. MitoSOX Staining

RSC96 cells were seeded at 1 × 10^4^ cells/well in a 96-well black plate and incubated with 25 mM or 150 mM glucose and different concentrations of SalA. After 24 h, 50 *μ*L of MitoSOX (Invitrogen, USA) and Hoechst 33342 were added to each well and incubated at 37°C for 30 min. After washing three times with PBS buffer, the photographs were taken immediately under the microscope (Nikon ECLIPSE Ti-U, Japan).

### 2.20. Measurement of Mitochondrial Membrane Potential

Mitochondrial membrane potential (MMP, Δ*ψm*) was measured with fluorescent dye Rhodamine 123. To detect the MMP level, RSC96 cells were seeded at 1 × 10^4^ cells/well in a 96-well black plate and incubated with 25 mM or 150 mM glucose and different concentrations of SalA. After 24 h, RSC96 cells were incubated with 1 *μ*M of Rhodamine 123 at 37°C for 10 min. After washing three times with PBS, the fluorescence intensity (excitation 488 nm, emission 535 nm) was detected using a SpectraMax M5 microplate reader (Molecular Devices, USA).

### 2.21. MitoTracker Staining

Content of mitochondria was determined by a MitoTracker Green FM kit (Invitrogen, USA) according to the manufacturer's instruction. Briefly, RSC96 cells were treated with 25 mM or 150 mM glucose and different concentrations of SalA. After 24 h, the MitoTracker Green staining solution preincubated at 37°C was added and incubated at 37°C for 30 min. Then, the cells were observed by laser confocal microscopy (Leica TCS SP8 X, Germany).

### 2.22. Measurement of ATP Production

The intracellular ATP level was measured by CellTiter-Glo® kit (Promega, USA) according to the manufacturer's instruction. Briefly, RSC96 cells were treated with 25 mM or 150 mM glucose and different concentrations of SalA for 24 h. Then, 100 *μ*L of a CellTiter-Glo reagent was added and mixed thoroughly. The cells were lysed by shaking for 2 min and allowed to stabilize the luminescent signal for 10 min. Chemiluminescence values (unit: RLUs) were detected using a SpectraMax M5 microplate reader (Molecular Devices, USA).

### 2.23. LibDock Docking

To perform the molecular docking studies, the program LibDock of Discovery Studio software (BIOVIA, USA) was used to simulate docking, which is a high throughput docking algorithm that positions catalyst-generated ligand conformations in the active sites of protein based on the polar interaction sites (hotspots). Keap1 binds to the Nrf2 transcription factor to promote its degradation. Since altering the conformation of Keap1 leads to nuclear translocation of Nrf2, the small molecule conformation of SalA was docked into the binding pocket of the Keap1 Kelch domain (PDB ID: 4IQK) to see the docking structure of each ligand conformation with compound 16 as the ligand [17], showing that the ligand molecule interacts with the hydrogen bond, hydrophobic bond, halogen bond, and Pi-Pi of the protein to obtain a two-dimensional plan of the ligand-protein interaction and scores according to the LibDock score to evaluate the affinity of the small molecule.

### 2.24. Reporter Assay

RSC96 cells were seeded at 5 × 10^4^ cells/well in a 24-well plate and then transfected with 0.5 *μ*g of a pGL4-ARE plasmid (Promega, USA) along with a promoterless Renilla luciferase construct using jetPRIME reagent (Polyplus, USA). 4 h after transfection, the reagent was removed and the cells were incubated with 25 mM or 150 mM glucose and different concentrations of SalA. Cell lysates were collected after 24 h treatment and firefly and Renilla luciferase activities were detected with a Dual-Glo Luciferase Assay System (Promega, USA).

### 2.25. Western Blotting

The cells were lysed in a RIPA buffer supplemented with complete protease inhibitor cocktail (CWBIO, China). Equal amount of protein was subjected to SDS-PAGE and immunoblotted with different antibodies. Multiple exposures were used to ascertain signal linearity. Quantitative analysis was measured using Gel-Pro software (Media Cybernetics, USA).

### 2.26. High Content Screening Assay

RSC96 cells were seeded at 1 × 10^4^ cells/well in a 96-well black plate and incubated with 25 mM or 150 mM glucose and SalA. After 24 h, the cells were fixed with 4% paraformaldehyde for 30 min following by 0.3% Triton to punch at room temperature for 30 min. After washing 3 times with PBS buffer and blocking with 5% BSA for 1 h, the cells were incubated with a primary antibody of anti-Nrf2 (1 : 500) overnight at 4°C followed by incubation of goat anti-mouse fluorescent secondary antibody (1 : 1000) for 1 h at room temperature. After washing three times with PBS, the cells were stained with Hoechst 33342 for 10 min and placed in a high content screening (HCS) system of operetta CLS (PerkinElmer, USA). The fluorescent image was taken and fluorescent intensity was analyzed. The translocation rate of Nrf2 was calculated automatically with its built-in program. Briefly, delineating the character region of cytosol and nuclear, the nuclear translocation rate was then calculated by the ratio of the intensity of nuclear and cytosol.

### 2.27. Knockdown of Nrf2 in RSC96 Cells

RSC96 cells were seeded at 5 × 10^4^ cells/well in a 24-well plate and then transfected with 110 pmol of Nrf2 SiRNA(r) (sc-156128, Santa Cruz, USA) using a jetPRIME reagent (Polyplus, USA). 24 h after transfection, the reagent was removed and the cells were cultured for another 24 h. Then the medium were changed to 25 mM or 150 mM glucose and different concentrations of SalA. After 24 h treatment, cell viability was detected with CCK-8 assay.

### 2.28. Statistical Analysis

All data were expressed as mean ± S.E.M. One-way analysis of variance (ANOVA) compared the mean values by GraphPad Prism 7 software (GraphPad Software Inc., CA, USA). For all comparisons, *P* value < 0.05 was considered statistically significant.

## 3. Results

### 3.1. Salvianolic Acid A Protected against High Glucose-Induced Injury in Rat RSC96 Schwann Cells

**Figure 1:**
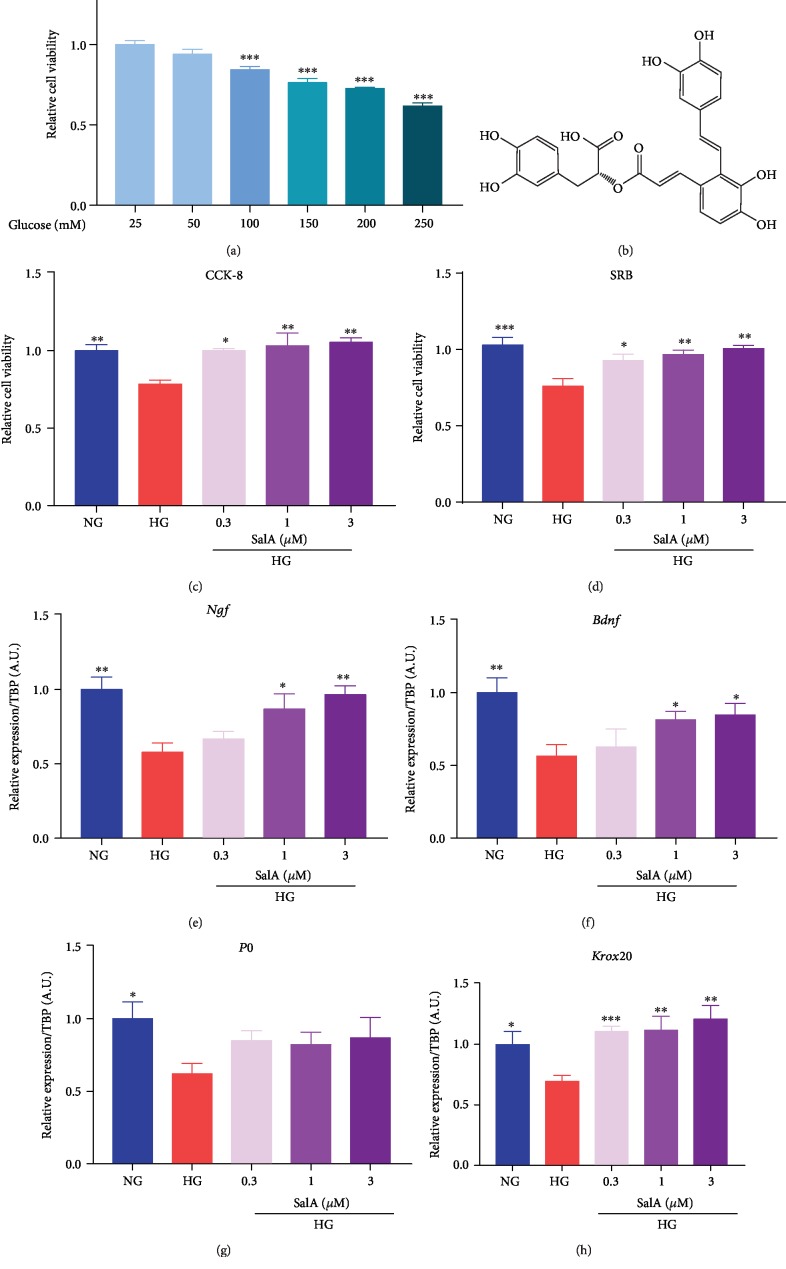
Salvianolic acid A protected against high glucose-induced injury in rat RSC96 Schwann cells. (a) After RSC96 cells were treated with various concentrations of glucose for 24 h, cell viability was measured by CCK-8 assay. All data are presented as mean ± SEM, *n* = 4. ^∗^*P* < 0.05 and ^∗∗∗^*P* < 0.001, compared with glucose 25 mM group. (b) Chemical structure of SalA. RSC96 cells were treated with 150 mM glucose and different concentrations of SalA for 24 h. Cell viability was measured by CCK-8 assay (c) and SRB assay (d). All data are presented as mean ± SEM, *n* = 4. ^∗^*P* < 0.05, ^∗∗^*P* < 0.01, and ^∗∗∗^*P* < 0.001, compared with the HG group. qPCR analysis of the *Ngf* (e), *Bdnf* (f), *P0* (g), and *Krox20* (h) gene expression in RSC96 cells treated with 150 mM glucose and different concentrations of SalA for 24 h. All qPCR data are normalized to TBP and presented as mean ± SEM, *n* = 3. ^∗^*P* < 0.05, ^∗∗^*P* < 0.01, and ^∗∗∗^*P* < 0.001, compared with the HG group.

Schwann cells (named after physiologist Theodor Schwann) are a variety of glial cells that keep peripheral nerve fibers alive. To evaluate the protective effects of SalA *in vitro*, we firstly treated rat RSC96 Schwann cells with the various concentrations of glucose to mimic the *in vitro* DPN model. We observed that 100-250 mM glucose significantly injured RSC96 cells in a dose-dependent manner with 24% reduction of cell viability at 150 mM glucose stimulation (Figure 1(a)). Thus, 150 mM glucose was chosen as the optimal injury condition for the following experiments. Meanwhile, we found that SalA (Figure 1(b)) treatment dose-dependently attenuated high glucose-induced injury of RSC96 cells which was detected using both CCK-8 (Figure 1(c)) and SRB assays (Figure 1(d)).

P0 and Krox20 play a role in maintaining the structure integrity of both myelin formation and axon. In addition, BDNF and NGF are important positive regulators of myelination secreted by Schwann cells [18]. Hence, we detected *Ngf*, *Bdnf*, *P0* and *Krox20* mRNA expression using a qPCR assay and observed that high glucose injury downregulated *Ngf*, *Bdnf*, *P0*, and *Krox20* mRNA expression, while SalA partially restored the downregulated genes expression (Figures 1(e)–1(h)) which is associated with myelination.

Taking together, we successfully established the *in vitro* DPN cell model for drug screening and evaluation and demonstrated that SalA mitigated the high glucose-induced injury in RSC96 cells, suggesting SalA might be potentially beneficial for treating diabetic peripheral neuropathy.

### 3.2. Effect of Salvianolic Acid A on the General Character in KK-Ay Diabetic Mice

**Figure 2:**
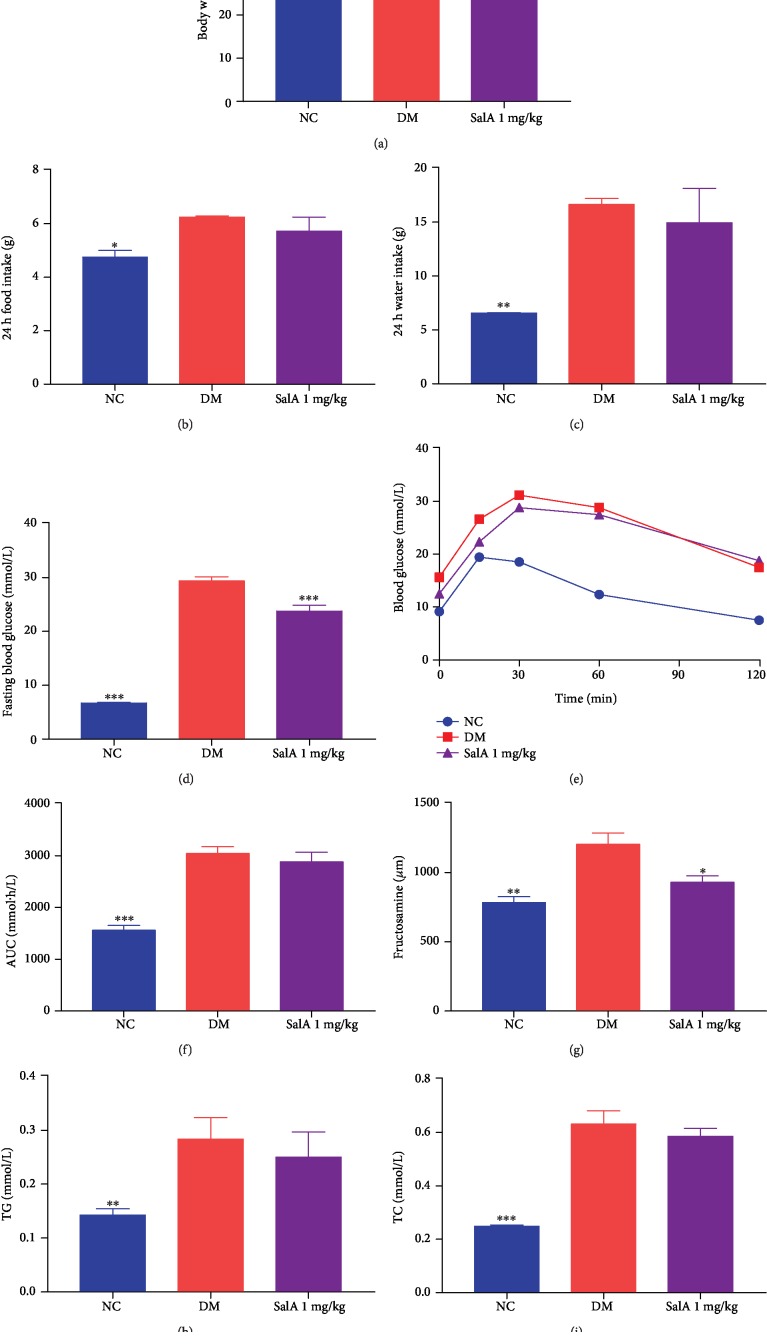
Salvianolic acid A ameliorated the general character in KK-Ay diabetic mice. SalA 1 mg/kg was orally administered to KK-Ay diabetic mice for 8 weeks and the general parameters of diabetic mice were measured. (a) Body weight, (b) 24 h food intake, (c) 24 h water intake, (d) fasting blood glucose, (e) GTT, (f) AUC of GTT, (g) serum level of fructosamine, (h) TG, and (i) TC were measured. All data are presented as mean ± SEM, *n* = 8‐10. ^∗^*P* < 0.05, ^∗∗^*P* < 0.01, ^∗∗∗^*P* < 0.001, compared with the DM group.

KK-Ay mice develop type 2 diabetes spontaneously which are obese, hyperglycemic, and hyperinsulinemic and are used for studying diabetes and diabetic complications. To further confirm the protective effects of SalA on diabetic peripheral neuropathy *in vivo*, we treated KK-Ay diabetic mice with oral administration of SalA. After 8-week treatment, though SalA barely influenced the body weight (Figure 2(a)), 24-food intake (Figure 2(b)), and 24-water intake (Figure 2(c)) of diabetic mice, it lowered 19.1% of fasting blood glucose significantly (Figure 2(d)) and tended to ameliorate the glucose tolerance determined by the area under the curve (AUC) after glucose loading yet lack of statistical significance (Figures 2(e) and 2(f)). The hypoglycemic effect of SalA was further verified with the reduction of fructosamine which is a marker of glucose control reflecting the average glycaemia level over the preceding 2-3 weeks (Figure 2(g)). Furthermore, SalA mildly reduced plasma 11.9% of TG (Figure 2(h)) and 7.2% of TC (Figure 2(i)) compared with KK-Ay mice without significant difference.

### 3.3. Salvianolic Acid A Protected against the Peripheral Neuropathy in KK-Ay Diabetic Mice

**Figure 3:**
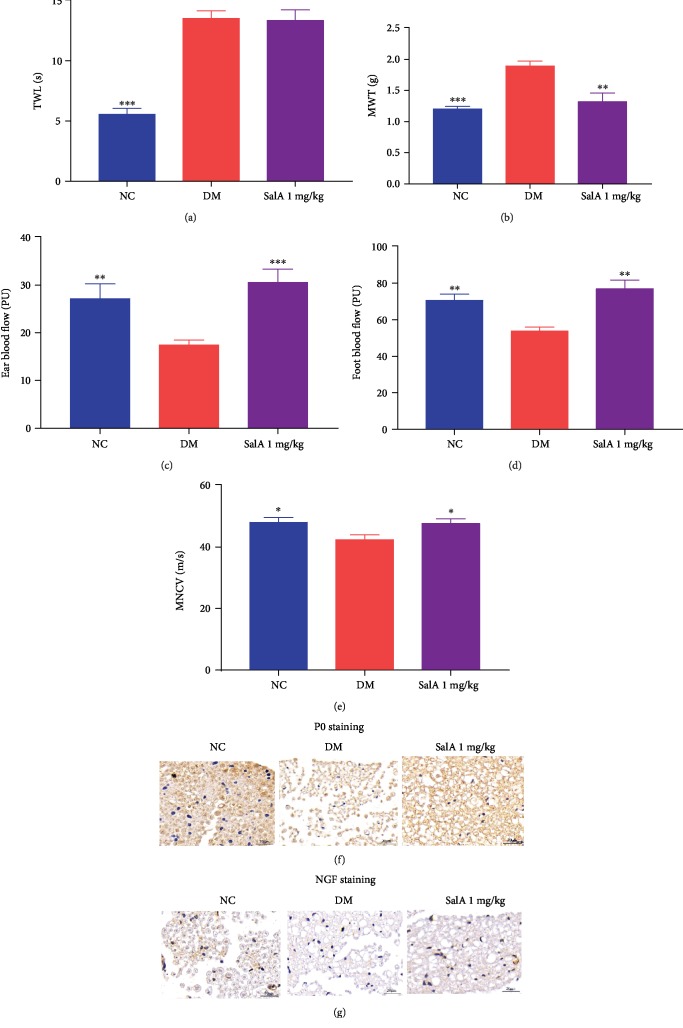
Salvianolic acid A protected against the peripheral neuropathy in KK-Ay diabetic mice. SalA 1 mg/kg was orally administered to KK-Ay diabetic mice for 8 weeks and the sciatic nerve function, including (a) thermal withdrawal latency (TWL), (b) mechanical withdrawal threshold (MWT), (c) ear blood flow, (d) foot blood flow, and (e) motor nerve conduction velocity (MNCV), was assessed. Representative images of immunohistological staining of P0- (f) and NGF- (g) positive cells from the NC, DM, and SalA 1 mg/kg groups (scale bar = 20 *μ*m). All data are presented as mean ± SEM, *n* = 8‐10. ^∗^*P* < 0.05, ^∗∗^*P* < 0.01, and ^∗∗∗^*P* < 0.001 compared with the DM group.

DPN is characterized as the impaired sensory and motor nerve function, especially the distal sensory nerve of the lower extremity. Mechanical allodynia and thermal hyperalgesia displayed as the elevated thermal withdrawal latency and mechanical withdrawal threshold combined with lower conduction velocities of sciatic nerve, which were observed in KK-Ay mice confirming the presence of impaired peripheral neuropathy. SalA administration markedly rescued the mechanical allodynia with the reduction of mechanical withdrawal threshold (Figure 3(b)) despite hardly affecting thermal hyperalgesia (Figure 3(a)). Concomitantly, SalA corrected the decreased ear and foot blood flow to nerves compared with diabetic mice (Figures 3(c) and 3(d)). More importantly, SalA increased the motor nerve conduction velocity of the sciatic nerve (Figure 3(e)). Furthermore, the upregulated P0 (Figure 3(f)) and NGF (Figure 3(g)) protein expression demonstrated by immunohistological staining reconfirmed the neuroprotective effects of SalA on peripheral neuropathy induced by diabetes.

Taken together, SalA protected against the diabetic peripheral neuropathy, especially rescuing the distal sensory loss in KK-Ay diabetic mice.

### 3.4. Salvianolic Acid A Restored the Ultrastructural Changes of the Injured Sciatic Nerve in KK-Ay Diabetic Mice

**Figure 4:**
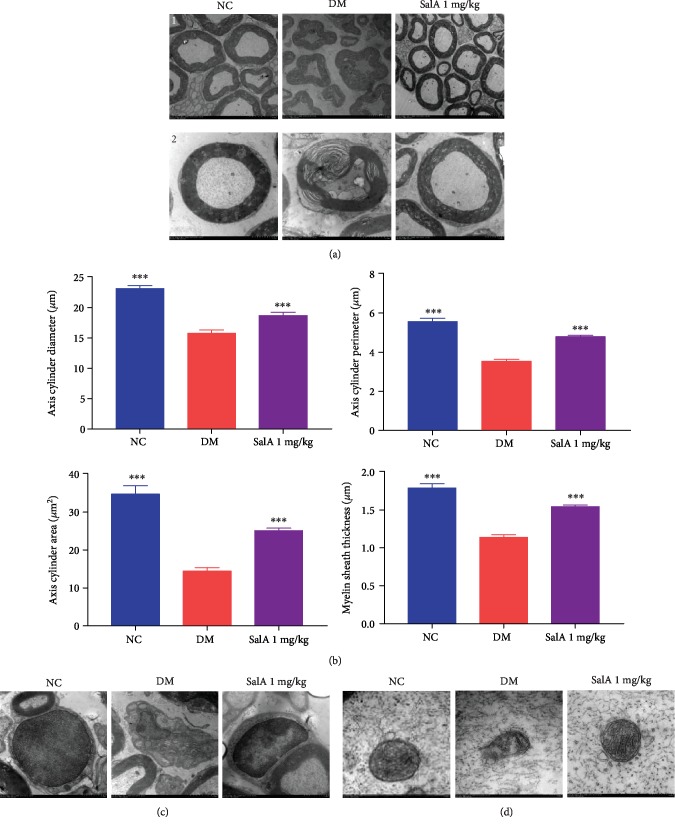
Salvianolic acid A restored the ultrastructural changes of the injured sciatic nerve in KK-Ay diabetic mice. SalA 1 mg/kg was orally administered to KK-Ay diabetic mice for 8 weeks and the ultrastructure of the sciatic nerve was detected by scanning transmission electron microscopy. (a) Representative ultrastructural images of the myelin sheath ((1): scale bar = 5 *μ*m; (2): scale bar = 2 *μ*m). (b) Quantitative analysis of diameter, perimeter and area of axis cylinder, and myelin sheath thickness. Representative ultrastructural images of Schwann cells (scale bar = 1 *μ*m) (c) and mitochondria (scale bar = 200 nm) (d) of the sciatic nerve from the NC, DM, and SalA 1 mg/kg groups. All data are presented as mean ± SEM, *n* = 8‐10. ^∗∗∗^*P* < 0.001 compared with the DM group.

To obtain the ultrastructural verification for the neuroprotective effects of SalA, we further utilized the scanning transmission electron microscopy to detect the ultrastructural changes of the injured sciatic nerve. Consistent with the previous literature, the myelin sheath and Schwann cells of the sciatic nerve were obviously destroyed by long-term hyperglycemia. However, SalA treatment partially restored the impaired myelin sheath (Figure 4(a)) with increased diameter, perimeter, area of axis cylinder, and myelin sheath thickness (Figure 4(b)). Schwann cells are responsible for maintaining structural and functional integrity of neurons and repairing the damaged nerves. As shown in Figure 4(c), the integrity of Schwann cells was damaged in the DM group and SalA administration markedly repaired the injured Schwann cells. As the power houses, mitochondria also underwent the relatively large changes in structure with swollen mitochondria and broken or disappeared mitochondria cristae under diabetic condition, whereas mitochondrial swelling shrank and broken mitochondrial cristae were ameliorated after SalA treatment, indicating the restored dynamic bioenergetic compartments (Figure 4(d)). Collectively, SalA restored the ultrastructural impairment and promoted the regeneration of myelin sheath, Schwann cells, and mitochondria of the injured sciatic nerve induced by diabetes.

### 3.5. Salvianolic Acid A Attenuated Oxidative Stress of RSC96 Schwann Cells under High Glucose Stimulation

**Figure 5:**
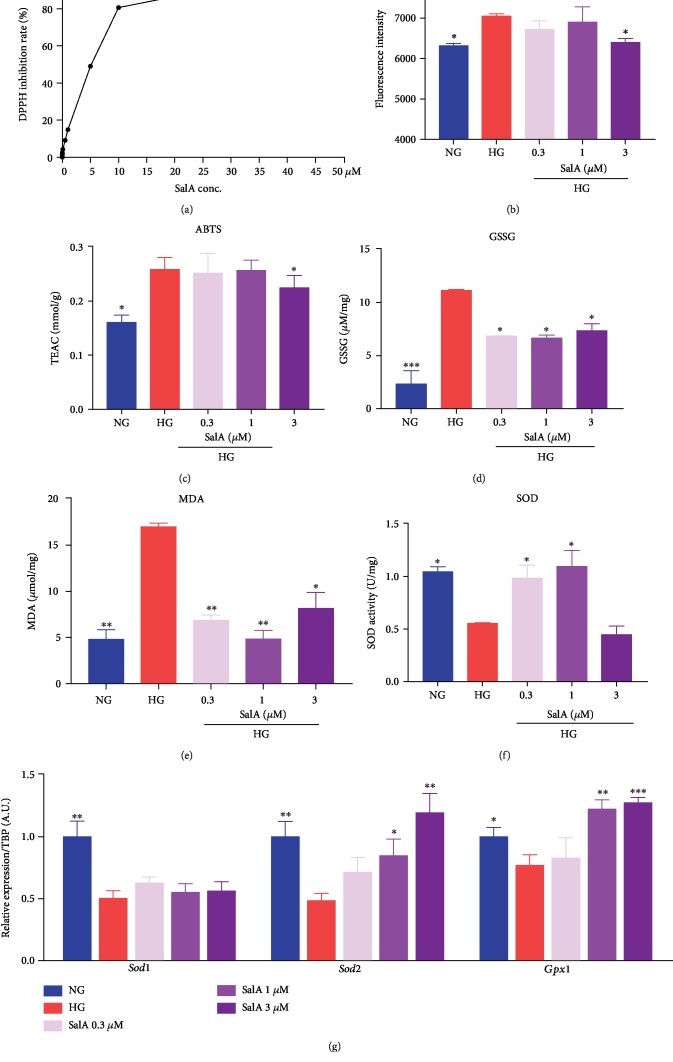
Salvianolic acid A attenuated oxidative stress in high glucose-injured RSC96 Schwann cells. (a) *In vitro* DPPH assay. Fresh DPPH and different concentrations of SalA were incubated at room temperature for 30 min. The absorbance value at 515 nm was recorded by a SpectraMax M5. RSC96 cells were treated with 150 mM glucose and different concentrations of SalA for 24 h. Intracellular ROS (b), ABTS (TEAC) (c), GSSG (d), MDA (e), and SOD activity (f) were measured using the corresponding kits. All data are presented as mean ± SEM, *n* = 3. ^∗^*P* < 0.05, ^∗∗^*P* < 0.01, and ^∗∗∗^*P* < 0.001, compared with the HG group. (g) qPCR analysis of the *Sod1*, *Sod2*, and *Gpx1* gene expression in RSC96 cells treated with 150 mM glucose and different concentrations of SalA for 24 h. All qPCR data are normalized to TBP and presented as mean ± SEM, *n* = 3. ^∗^*P* < 0.05, ^∗∗^*P* < 0.01, and ^∗∗∗^*P* < 0.001, compared with the HG group.

To further investigate the protective mechanism of SalA, we firstly determined oxidative stress, which is believed to be closely involved in the persistent hyperglycemia-induced injury in Schwann cells. Firstly, we performed an *in vitro* DPPH inhibition assay to check the antioxidative activity of SalA and found that SalA (10^−10^-10^−5^ M) exhibited DPPH radical scavenging activity in a dose-dependent manner, indicating that SalA exhibited strong antioxidative activity (Figure 5(a)). Additionally, we further detected the balance of free radicals and antioxidative ability to evaluate HG-induced oxidative stress and the antioxidative stress effects of SalA. As presented in Figure 5(b), high glucose stimulation elevated the intracellular ROS production which causes damage to lipids, proteins, and DNA in oxidative stress. As a strong antioxidant, SalA reduced ROS production in a dose-dependent manner. Likewise, SalA reduced the cellular content of ABTS which is the substrate of catalase (CAT) as the trolox equivalent antioxidant capacity (TEAC) (Figure 5(c)) and markedly lowered the GSSG concentration (Figure 5(d)) and MDA content (Figure 5(e)), which are considered as the important markers of oxidative stress. As one of typical antioxidative enzymes, total SOD activity was reduced by high glucose injury and restored under 0.3 and 1 *μ*M of SalA treatment, instead of 3 *μ*M, which needs further investigation (Figure 5(f)). Finally, we further determined the gene expression of antioxidant enzymes including SOD1, SOD2, and glutathione peroxidase 1 (GPX1) and found that high glucose challenge indeed impaired antioxidative ability with the downregulated mRNA expression of *Sod1*, *Sod2*, and *Gpx1*. SalA treatment corrected the *Sod2* and *Gpx1* expression but without any alteration in *Sod1* mRNA expression (Figure 5(g)). Mitochondrial superoxide dismutase SOD2, instead of cytosolic SOD1, protects against glutamate-induced oxidative stress [19]. Thus, our findings indicated that SalA might exert the antioxidative effects more in mitochondria than in cytoplasm.

Collectively, SalA attenuated oxidative stress of RSC96 cells associated with high glucose-induced injury.

### 3.6. Salvianolic Acid A Mitigated Neuroinflammation of RSC96 Schwann Cells Injured by High Glucose Challenge

**Figure 6:**
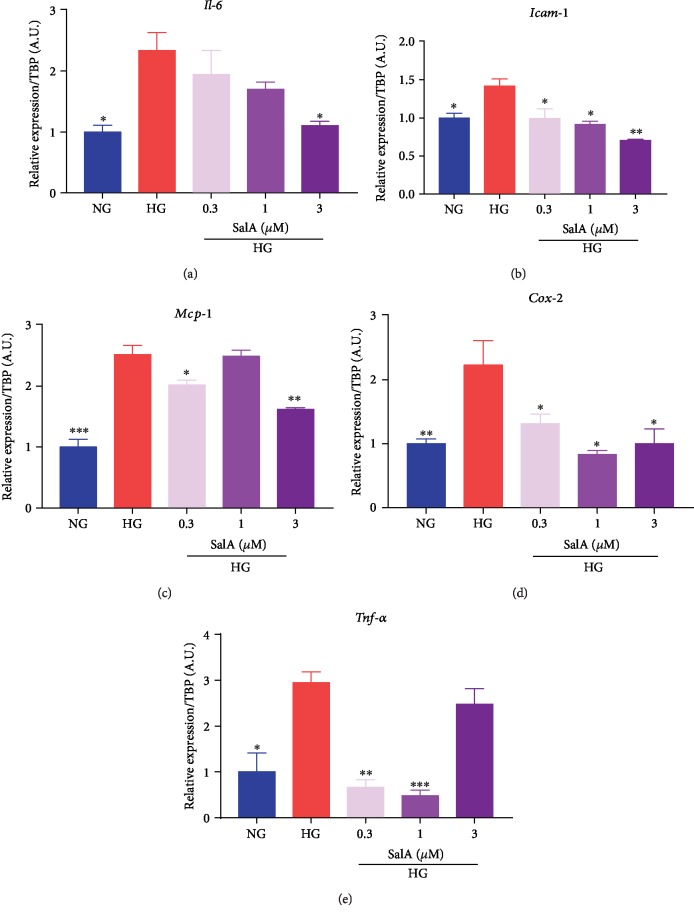
Salvianolic acid A mitigated neuroinflammation in RSC96 Schwann cells injured by high glucose. RSC96 cells were treated with 150 mM glucose and different concentrations of SalA for 24 h. qPCR analysis of the *Il-6* (a), *Icam-1* (b), *Mcp-1* (c), *Cox-2* (d), and *Tnf-α* (e) gene expression was performed. All qPCR data are normalized to TBP and presented as mean ± SEM, *n* = 3. ^∗^*P* < 0.05, ^∗∗^*P* < 0.01, and ^∗∗∗^*P* < 0.001, compared with the HG group.

Neuroinflammation is demonstrated to be modulated by oxidative stress and inflammatory cytokines, such as interleukin-6 (IL-6), tumor necrosis factor-*α* (TNF-*α*), and nuclear factor-kappa B (NF-*κ*B), which may cause progressive myelin sheath damage. Thus, to further investigate the protective mechanism of SalA, we determined the neuroinflammation in HG-injured Schwann cells with the detection of inflammatory factor expression. Utilizing qPCR assay, we found that high glucose injury upregulated the inflammatory factors mRNA expression of *Il-6* (Figure 6(a)), *intercellular adhesion molecule 1* (*Icam-1*) (Figure 6(b)), *monocyte chemoattractant protein-1* (*Mcp-1*) (Figure 6(c)), *cyclooxygenase-2* (*Cox-2*) (Figure 6(d)), and *Tnf-α* (Figure 6(e)), indicating that high glucose stimulation induced the distinct neuroinflammation in RSC96 Schwann cells, whereas SalA treatment significantly downregulated the inflammatory factor expression of *Il-6* (Figure 6(a)), *Icam-1* (Figure 6(b)), *Mcp-1*(Figure 6(c)), *Cox-2* (Figure 6(d)), and *Tnf-α* mRNA (Figure 6(e)). Overall, SalA mitigated neuroinflammation of RSC96 cells induced by high glucose challenge.

### 3.7. Salvianolic Acid A Improved the Mitochondrial Function in High Glucose-Injured RSC96 Schwann Cells

**Figure 7:**
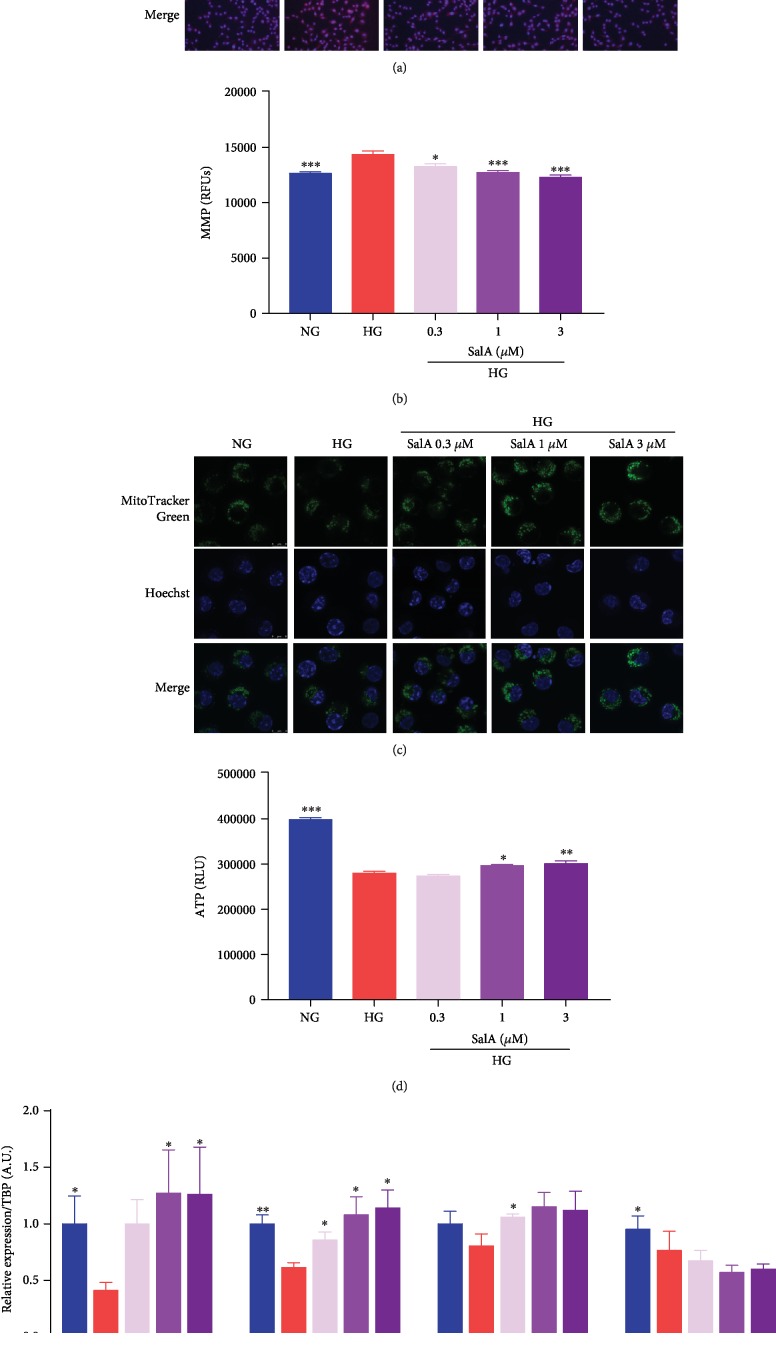
Salvianolic acid A improved the mitochondrial function in high glucose-injured RSC96 Schwann cells. RSC96 cells were treated with 150 mM glucose and different concentrations of SalA for 24 h. (a) Representative images showing fluorescence of mitochondrial superoxide were detected by MitoSOX™ Red staining (scale bar = 50 *μ*m). (b) Mitochondrial membrane potential was examined after the cells were incubated with fluorescent dye Rhodamine 123 for 10 min. The fluorescence intensity (excitation 488 nm, emission 535 nm) was detected using SpectraMax M5 microplate reader. (c) Mitochondrial morphology visualized by staining with MitoTracker Green FM was examined using a laser scanning microscope (scale bar = 8 *μ*m). (d) Intracellular ATP level was determined by a CellTiter-Glo® kit. All data are presented as mean ± SEM, *n* = 3. ^∗^*P* < 0.05, ^∗∗^*P* < 0.01, and ^∗∗∗^*P* < 0.001, compared with the HG group. (e) qPCR analysis of the *Cox8b*, *Sdhb*, *Atp5j*, and *Uqcrc1* gene expression in RSC96 cells treated with 150 mM glucose and different concentrations of SalA for 24 h. All qPCR data are normalized to TBP and presented as mean ± SEM, *n* = 3. ^∗^*P* < 0.05 and ^∗∗^*P* < 0.01, compared with the HG group.

To explore whether SalA targeted the mitochondrial function, we firstly investigated the effects of SalA on mitochondrial ROS production in HG-injured RSC96 cells. As a red mitochondrial superoxide indicator, MitoSOX-based fluorescent staining can reliably detect the differences in mitochondrial ROS formation in cells. Apparently, high glucose injury induced more mitochondrial ROS than the NG group with the highly fluorescent exhibition. SalA incubation markedly reversed the stronger MitoSOX staining (red fluorescence) of RSC96 cells in the HG group, revealing that SalA scavenged the mitochondrial ROS formation induced by high glucose injury (Figure 7(a)). Measurement of mitochondrial membrane potential in living cells can be used to assess the mitochondrial function. A lower membrane potential can prevent free radical production. So, we investigated whether SalA affected MMP using Rhodamine 123 fluorescence staining. Interestingly, we observed that SalA treatment dose-dependently reduced Rhodamine 123 fluorescence in high glucose-treated RSC96 cells, suggesting that SalA decreased MMP (Figure 7(b)). MitoTracker Green FM is a green-fluorescent mitochondrial stain localizing to mitochondria for mitochondrial mass estimation, regardless of MMP. We observed a decreased MitoTracker Green fluorescence after high glucose injury, whereas SalA markedly boosted the fluorescence, reflecting the enhanced mitochondrial content (Figure 7(c)). ATP production is the main activity of mitochondria. As a result, we observed that ATP production displayed significant reduction after 150 mM of high glucose injury, whereas SalA dose-dependently promoted ATP production of RSC96 cells in mitochondria (Figure 7(d)). *Cox8b*, *Sdhb*, and *Uqcrc1* participate in the mitochondrial respiratory chain, and *Atp5j* catalyzes ATP synthesis using an electrochemical gradient of protons across the inner membrane during oxidative phosphorylation. As we expected, SalA upregulated the mitochondrial gene expression of *Cox8b*, *Sdhb*, and *Atp5j* in a dose-dependent manner to improve the mitochondrial oxidative phosphorylation (Figure 7(e)).

Altogether, SalA scavenged the mitochondrial ROS, decreased MMP, enhanced mitochondrial content and ATP production, upregulated the oxidative phosphorylation gene expression, and finally improved mitochondrial function of RSC96 Schwann cells injured by high glucose, consistent with the mitochondria-protective effects of SalA in KK-Ay mice (Figure 4).

### 3.8. Salvianolic Acid A Exerted the Protective Effect on High Glucose-Injured RSC96 Cells through Modulation of Nrf2

**Figure 8:**
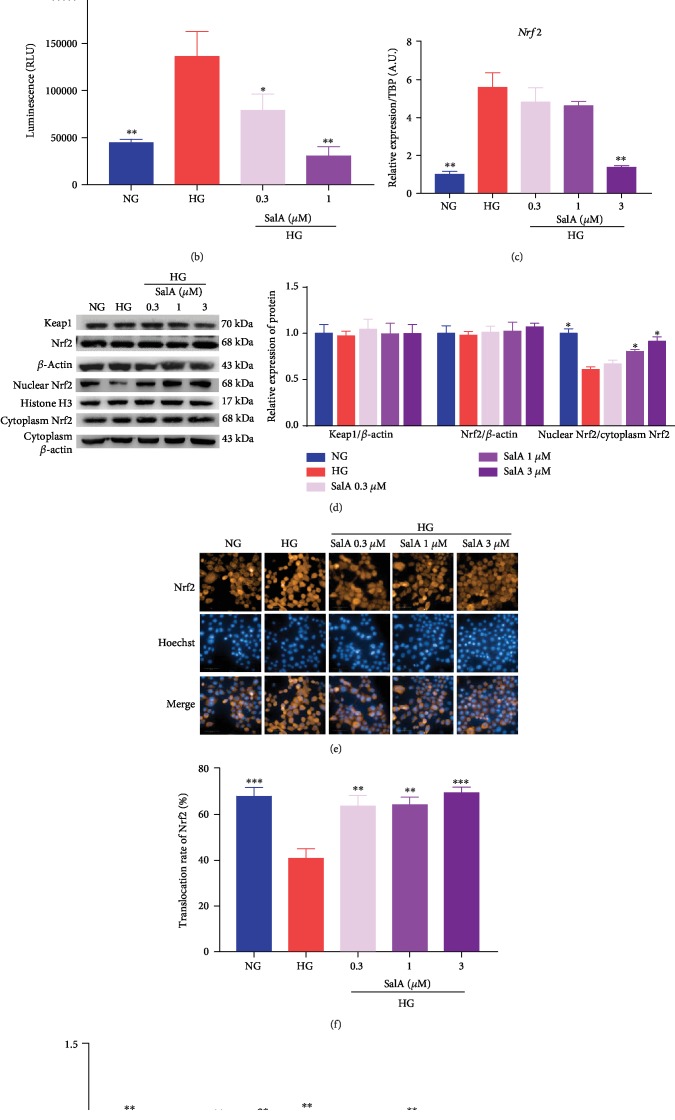
Salvianolic acid A exerted the protective effect on high glucose-injured RSC96 cells through modulation of Nrf2. (a) Molecular interaction of SalA with the active sites of the Keap1 Kelch domain using the program LibDock of Discovery Studio software. (b) RSC96 cells were transfected with pGL4-ARE along with a promoterless Renilla luciferase construct using the jetPRIME reagent and then treated with 150 mM glucose and different concentrations of SalA. The promoter activity of luciferase was detected by a reporter assay kit. All data are presented as mean ± SEM, *n* = 4. ^∗^*P* < 0.05, ^∗∗^*P* < 0.01, and ^∗∗∗^*P* < 0.001, compared with the HG group. (c) qPCR analysis of the *Nrf2* gene expression in RSC96 cells treated with 150 mM glucose and different concentrations of SalA for 24 h. All qPCR data are normalized to TBP and presented as mean ± SEM, *n* = 3. ^∗^*P* < 0.05, ^∗∗^*P* < 0.01, ^∗∗∗^*P* < 0.001, compared with the HG group. (d) Representative blot image and quantitative analysis (*n* = 3 western blot) of total Keap1, total Nrf2, total *β*-actin (loading control), nuclear Nrf2, histone H3 (loading control), cytoplasm Nrf2, and cytoplasm *β*-actin (loading control) protein in RSC96 cells treated with 150 mM glucose and different concentrations of SalA for 24 h from independent experiments. RSC96 cells were treated with 150 mM glucose and different concentrations of SalA, followed by staining using Nrf2 antibody. The cells were scanned by a high content screening assay (scale bar = 50 *μ*m) (e), and average relative fluorescence was quantified by the intensity of fluorescence from individual cells (f). (g) RSC96 cells were transfected with control SiRNA or Nrf2 SiRNA using a jetPRIME reagent. 48 h after transfection, the cells were incubated with 150 mM glucose and different concentrations of SalA. After 24 h treatment, the cell viability was detected with a CCK-8 assay. All data are presented as mean ± SEM, *n* = 3. ^∗^*P* < 0.05, ^∗∗^*P* < 0.01, and ^∗∗∗^*P* < 0.001, compared with the HG group.

To identify the potential regulator mediating the protective effects of SalA, we firstly explored the key transcript factor of Nrf2, which is involved in oxidative stress and mitochondria. Firstly, molecular docking was performed to predict the potential interaction of SalA and Keap1/Nrf2 using the program LibDock of Discovery Studio software. The docking results demonstrated that SalA was docked into active sites of Keap1 Kelch domain at ARG483, ARG415, ARG380, GLY364, SER363, GLY603, TYR334, SER602, ALA556, GLY509, and ILE461 and showed good LibDock interaction energy (LibDock score = 171.891), higher than ligand-compound 16 (LibDock score = 151.089, RMSD 2.1105) [17]. It suggested that SalA is directly bound to the Kelch domain of Keap1 through the hydrogen bond, Pi bond, and van der Waal interaction, and thus disrupted the interaction of Nrf2 and Keap1, finally influencing the Nrf2 expression (Figure 8(a)). To verify the relationship between SalA and Nrf2, we first need to know whether SalA affects Nrf2 promoter activity; therefore, a pGL4-ARE plasmid was used for the reporter assay. After transfecting RSC96 cells with the pGL4-ARE plasmid, we surprisingly observed that the Nrf2 promoter activity was enhanced by high glucose stimulation, but SalA treatment significantly inhibited the Nrf2 promoter activity with a lower luminescence value (Figure 8(b)). Consistently, SalA treatment distinctly downregulated Nrf2 mRNA expression, especially 3 *μ*M of SalA (Figure 8(c)). Interestingly, at the translation stage, neither upregulation nor downregulation was detected in Nrf2 and upstream Keap1 protein expression using western blotting assay (Figure 8(d)).

Additionally, we were also interested in how SalA affected Nrf2 translocation from cytoplasm to the nucleus. Contrary to the effects on transcription stage, Nrf2 nuclear translocation was inhibited by 150 mM of high glucose, whereas SalA significantly upregulated nuclear Nrf2 expression and enhanced Nrf2 nuclear translocation dose-dependently with the strongest effect of SalA 3 *μ*M using western blotting assay and high content screening assay (Figures 8(d)–8(f)). Finally, to validate that SalA-induced Nrf2 cellular localization was involved in its antiglucotoxicity effect, we tested the protective effect of SalA in Nrf2 knockdown cells. After Nrf2 was knocked down in RSC96 cells using Nrf2 SiRNA, we found that SalA could not protect the RSC96 cells against the injury induced by high glucose compared with the control SiRNA, confirming that Nrf2 mediated the protective effects of SalA on diabetic peripheral neuropathy (Figure 8(g)).

Taken together, it could be inferred that SalA exerted the protective effects on HG-injured RSC96 cells through modulation of Nrf2.

## 4. Discussion

The current data presented here clearly demonstrated that high glucose-injured rat RSC96 Schwann cells induced dramatical oxidative stress, neuroinflammation, and mitochondrial dysfunction and finally led to peripheral nerve injury showed by the downregulated myelin proteins and transcription factor expression. Salvianolic acid A exerted the protective effects on Schwann cells against high glucose-induced injury through modulation of a transcript factor Nrf2. More importantly, SalA ameliorated the impaired peripheral nerve function in type 2 diabetic mice with upregulated mechanical withdrawal threshold and increased blood flow and nerve conduction velocity.The beneficial effects of SalA were further confirmed by restoring the ultrastructural changes of the injured sciatic nerve.

Diabetic peripheral neuropathy is characterized by diffuse damage to peripheral nerve fibers which causes the debilitating pain and sensory loss [2]. Hyperglycemia­induced excess intracellular glucose is mainly removed by glycolysis, however, excess pyruvate from glycolysis is thought to injure the neurons. In addition, aldose reductase reduces glucose to sorbitol which is oxidized to fructose by sorbitol dehydrogenase [20], leading to the increased cellular osmolarity and the depleted nicotinamide adenine dinucleotide phosphate (NADPH), finally leading to oxidative stress [21, 22]. Oxidative stress damages the mitochondria and other cellular components of neurons, microvascular endothelial cells, and Schwann cells, contributing to the pathogenesis of neuropathy.

Additionally, persistent hyperglycemia is believed to induce neuroinflammation and neuronal damage as the glycosylation of myelin protein causes monocyte, neutrophil and macrophage infiltration, which in turn secrete the inflammatory cytokines, such as IL-6, TNF-*α*, and NF-*κ*B, to further damage myelin sheath and increase nerve excitability, thus leading to edema and neuroinflammation [23, 24]. Neuroinflammation is also demonstrated to be modulated by oxidative stress. In a redox balance, the inflammation is a beneficial defense. However, if oxidative stress persistently exists, the inflammation will cause the progressive tissue damage. Recent findings showed that Morin and natural rotenoid deguelin exerted neuroprotection in experimental diabetic neuropathy through antioxidative stress and antineuroinflammation [7, 25]. Thus, targeting the cellular oxidative stress and inflammation pathway will be likely important therapeutic approaches in the future to reduce the incidence of diabetic neuropathy.

On the basis of this, we established a high glucose-injured cellular model using rat RSC96 cells to mimic *in vitro* diabetic peripheral neuropathy model. We observed that high glucose dose-dependently injured the cell viability of RSC96, suggesting that the DPN cell model was successfully made. SalA, as a strong antioxidant agent, protected against the impaired cell viability and upregulated myelin proteins and transcription factor expression as well. Concomitantly, SalA attenuated oxidative stress and neuroinflammation induced by high glucose injury, finally protected against oxidative stress and inflammation-mediated neuronal damage.

Mitochondrial dysfunction has been implicated as a pathogenic factor of DPN. Several researches have illustrated that the mitochondrial respiratory chain has the reduced activity in dorsal root ganglia in type 1 and type 2 diabetic animal models [26, 27]. Mitochondrial functional abnormalities and alteration of the AMPK/SIRT/PGCl*α* pathway in Schwann cells are also implicated [28]. In addition to the disturbed functionality, the mitochondrial dynamics (size, shape, and number) is also known to affect the axon transfer, leading to various alterations of sensorimotor function. The glove and stocking patterns of thermal sensitivity are due to impairment of anterograde axonal transport in sensory neurons [29, 30]. Indeed, our experimental observation suggested that mitochondrial dysfunction of RSC96 cells was exacerbated by high glucose injury; also, mitochondrial ultrastructure was also impaired dramatically in the sciatic nerve of KK-Ay diabetic mice. Since mitochondria are the primary source of superoxide, SalA attenuated oxidative damage by scavenging the mitochondrial ROS formation and decreasing MMP. In addition, SalA upregulated the gene expression associated with mitochondrial oxidative phosphorylation, as well as increased mitochondrial content and ATP production, consequently improved mitochondrial function to alleviate the bioenergetic crisis associated with DPN, suggesting SalA is a mitochondrial antioxidant. Moreover, the previous studies of SalA in our lab have demonstrated that the regulation of SalA on glucose metabolism was largely related with AMPK activation and mitochondrial regulation [14]. The protective effect of SalA on DPN was closely related with its antidiabetic effect. Chronic stimulation of high glucose initiated the onset of peripheral neuropathy; thus, the antidiabetic of SalA was another mechanism of its protection against DPN besides the amelioration of high-glucose injury in Schwann cells.

Nrf2 is a double-edged sword. On the one hand, Nrf2 is a relatively unstable protein that regulates the expression of various antioxidant proteins (such as detoxifying enzymes) via ARE binding site. Consequently, Nrf2 is a master regulator of antioxidative responses. On the other hand, Nrf2 subverts the redox homeostasis during the evolution of cancer and promotes tumor maintenance [31–33]. Hence, drug discovery targeting Nrf2 by activation or inhibition is facing a dilemma.

Hyperglycemia-mediated oxidative stress also activates the cellular pathways like Nrf2 and NF-*κ*B. Activation of the Nrf2 pathway enhances the antioxidative activity to protect cells from the enhanced oxidative stress as one of the cellular homeostatic mechanisms [34]. However, persistent Nrf2 activation is subdued through hyperglycemia-mediated ERK activation, and thus redox homeostasis is disturbed in the diabetic state [35]. In our current study, we tried to clarify whether the antioxidative and anti-inflammatory effects of SalA were modulated by Nrf2 and surprisingly found the inconsistent results of Nrf2 at the different stages. Nrf2 promoter activity was induced, and Nrf2 mRNA was upregulated by high glucose stimulation, whereas Nrf2 protein expression was not altered and Nrf2 nuclear translocation was dramatically inhibited. Thus, we speculated that there might be posttranscriptional modification leading to the different modulation of Nrf2 at the different stages.

It is reported that SalA is a potential Nrf2 modulator in treating diabetes-associated macrovascular and renal injury [36]. We also found that SalA treatment could significantly inhibit Nrf2 promoter activity, downregulate Nrf2 mRNA expression, but promote Nrf2 nuclear translocation. Further knockdown of Nrf2 validated that SalA-induced Nrf2 cellular localization was involved in its antiglucotoxicity effect. Given that docking results demonstrated that SalA directly bound to Keap1 Kelch domain at multiple active sites, we speculated that SalA disrupted the interaction of Keap1 and Nrf2, subsequently promoted the nuclear translocation of Nrf2 leading to Nrf2 activation, and finally mediated the protective effects of SalA. Hence, our findings suggested that SalA protected against diabetic peripheral neuropathy through antioxidation and anti-inflammation modulated by Nrf2.

As of yet, there is no established treatment for therapeutic interventions of DPN in addition to glycemic control. As an aldose reductase inhibitor, Epalrestat has been used to treat diabetic peripheral neuropathy in the clinic for many years, whereas a very recent study reported that Epalrestat induced oxidative stress, inflammation, and fibrogenesis in the mouse liver [37].

## 5. Conclusions

In conclusion, SalA could effectively improve the abnormal glucose and lipid metabolism, significantly protected against diabetic peripheral neuropathy. Its underlying mechanisms may be involved in antioxidative stress, anti-inflammation, and improvement of mitochondrial function modulated by transcript factor Nrf2. In view of the excellent safety in toxicity experiments, SalA may be the promising clinical application for the treatment of diabetic peripheral neuropathy patients.

## Figures and Tables

**Table 1:** Primer sequences of genes confirmed with qPCR.

Gene		Primer sequence
rNgf	Forward primer:	5′- AAGGACGCAGCTTTCTATCC-3'
	Reverse primer:	5′- TTGCTATCTGTGTACGGTTCTG-3'

rBdnf	Forward primer:	5′- TGGCTCTCATACCCACTAAGA-3'
	Reverse primer:	5′- CGGAAACAGAACGAACAGAAAC-3'

rMpz/P0	Forward primer:	5′- ACAGGGAAAGCCAATGAGAATA-3'
	Reverse primer:	5′- GGACATTTGGAGATGGTAGGAG-3'

rKrox20	Forward primer:	5′- GTCGGGAAGGACAAAGCAATA-3'
	Reverse primer:	5′- CGGAGAACAGTGCACATCAA-3'

rSod1	Forward primer:	5′- TGGTGGTCCACGAGAAACAA-3'
	Reverse primer:	5′- TGGGCAATCCCAATCACACC-3'

rSod2	Forward primer:	5′- GCCTCAGCAATGTTGTGTCG-3'
	Reverse primer:	5′- ATTGTTCACGTAGGTCGCGT-3'

rGpx1	Forward primer:	5′- GTAGGTCCAGACGGTGTTCC-3'
	Reverse primer:	5′- ATCGGGTTCGATGTCGATGG-3'

rIL-6	Forward primer:	5′- TCTCTCCGCAAGAGACTTCCA-3'
	Reverse primer:	5′- ATACTGGTCTGTTGTGGGTGG-3'

rIcam-1	Forward primer:	5′- GCCTGGGGTTGGAGACTAAC-3'
	Reverse primer:	5′- CTCGCTCTGGGAACGAATACA-3'

rMcp-1	Forward primer:	5′- TCCACCACTATGCAGGTCTC-3'
	Reverse primer:	5′- GGGCATTAACTGCATCTGGCT-3'

rCox-2	Forward primer:	5′- AAGGCGTTCAACTGAGCTGT-3'
	Reverse primer:	5′- ACACAGGAATCTTCACAAATGGA-3'

rTNF-*α*	Forward primer:	5′- ATGGGCTCCCTCTCATCAGT-3'
	Reverse primer:	5′- GCTTGGTGGTTTGCTACGAC-3'

rCox8b	Forward primer:	5′- CCCGAGAAGTACACAGTGATT-3'
	Reverse primer:	5′- GAAGCGTTTAATTGGCCTCTC-3'

rSdhb	Forward primer:	5′- GAGGGCAAGCAACAGTATCT-3'
	Reverse primer:	5′- CTTGTAGGTCGCCATCATCTT-3'

rAtp5j	Forward primer:	5′- TCAGCAGTCTCTGTGCATTT-3'
	Reverse primer:	5′- ACTTATCCATCTCTCCTTTACCATAC-3'

rUqcrc1	Forward primer:	5′- CGCACAGACTTGACTGACTAC-3'
	Reverse primer:	5′- CACTGCTGGACATTGGTCATA-3'

rNrf2	Forward primer:	5′- GTACAACCCTTGTCACCATCTC-3'
	Reverse primer:	5′- TCCGATGACCAGGACTTACA-3'

rTbp	Forward primer:	5′- GAGAATAAGAGAGCCACGAACA-3'
	Reverse primer:	5′- TCTTGCTGCTAGTCTGGATTG-3'

## Data Availability

The data used to support the findings of this study are available from the corresponding author upon request.
